# Anti-angiogenesis therapy and gap junction inhibition reduce MDA-MB-231 breast cancer cell invasion and metastasis *in vitro* and *in vivo*

**DOI:** 10.1038/srep12598

**Published:** 2015-07-28

**Authors:** Kazem Zibara, Zahraa Awada, Leila Dib, Jamal El-Saghir, Sara Al-Ghadban, Aida Ibrik, Nabil El-Zein, Marwan El-Sabban

**Affiliations:** 1 ER045 - Laboratory of Stem Cells, PRASE, Biology Department, Faculty of Sciences, Lebanese University, Beirut, Lebanon; 2 Department of Anatomy, Cell Biology and Physiological Sciences, Faculty of Medicine, American University of Beirut, Beirut, Lebanon

## Abstract

Cancer cells secrete VEGF, which plays a key role in their growth, invasion, extravasation and metastasis. Direct cancer cell-endothelial cell interaction, mediated by gap junctions, is of critical importance in the extravasation process. In this study, we evaluated avastin (Av), an anti-VEGF antibody; and oleamide (OL), a gap junction inhibitor, using MDA-MB-231 human breast cancer cells *in vitro* and a xenograft murine model *in vivo*. Results showed that Av/OL significantly decreased proliferation, induced cell cycle arrest and decreased migration and invasion of MDA-MB-231 cells *in vitro*. In addition, Av/OL significantly decreased homo and hetero-cellular communication interaction between MDA-MDA and MDA-endothelial cells, respectively. The expression levels of several factors including VEGF, HIF1α, CXCR4, Cx26, Cx43, and MMP9 were attenuated upon Av/OL treatment *in vitro*. On the other hand, avastin, but not oleamide, reduced tumor size of NSG mice injected subdermally (*s.d.*) with MDA-MB-231 cells, which was also associated with increased survival. Furthermore, Av but also OL, separately, significantly increased the survival rate, and reduced pulmonary and hepatic metastatic foci, of intravenously (*i.v.*) injected mice. Finally, OL reduced MMP9 protein expression levels, better than Av and in comparisons to control, in the lungs of MDA-MB-231 i.v. injected NSG mice. In conclusion, while avastin has anti-angiogenic, anti-tumor and anti-metastatic activities, oleamide has anti-metastatic activity, presumably at the extravasation level, providing further evidence for the role of gap junction intercellular communication (GJIC) in cancer cell extravasation.

Metastasis accounts for more than 90% of cancer-related mortality. Metastatic colonization is a multistep process by which cancer cells detach from the primary tumor, invade the surrounding microenvironment, intravasate into pre-existing or newly formed blood vessels, survive the hemodynamics of circulation and the host immune system and extravasate at specific sites where they colonize secondary organs and generate distant metastases^1^,^2^. Cell-cell and cell-matrix interactions, angiogenesis, epithelial-mesenchymal transition (EMT), and soluble factors are involved in the progression and dissemination of cancer cells^1^,^2^. Tumor microenvironment, such as extracellular matrix, macrophages, fibroblasts, stem cells, and endothelial cells (ECs) are known to impact tumor growth and metastasis^3^,^4^. Tumor cells communicate with ECs, at the primary site or at the extravasation site, through paracrine mechanisms or through gap junctions (GJs) that directly link the cytoplasms of the two cells. Gap junctions are clusters of channels made up of connexin proteins that play a crucial role in tissue homeostasis, cell growth, differentiation and carcinogenesis^5^.

We have previously demonstrated that cancer cells communicate with endothelial cells of target organs through both paracrine stimulation and direct hetero-cellular communication, causing a breach in the endothelial barrier allowing cancer cell extravasation^6^,^7^. In breast cancers, several studies highlighted the role of connexins as tumor suppressor genes. In fact, studies have demonstrated that over-expression of connexins in breast cancer cells suppressed tumor growth *in vivo* and inhibited their malignant properties^8^,^9^.

Because of the role of GJs in cancer cell extravasation, a new promising anti-cancer approach was proposed by targeting extravasation, a crucial step in the metastatic process^10^. In this study, we evaluated the effect of heterocellular communication, mediated by cell-cell adhesion and gap junction formation, and the effect of paracrine secretion on extravasation, using MDA-MB-231 human breast cancer cells *in vitro* and a xenograft murine model *in vivo*.

## Materials and Methods

### Drugs

Bevacizumab (Avastin, Genentech, USA), an anti-VEGF monoclonal antibody, was used at a concentration of 50 μM, as described by Emlet *et al.*^11^. Oleamide (OL, Sigma, USA), a gap junction inhibitor, was prepared fresh from a stock solution of 10 mM dissolved in Dimethyl Sulfoxide (DMSO), diluted in culture media for *in vitro* assays, and used at 20 μM as determined by cytotoxicity assays (data not shown). Oleamide was dissolved fresh in sterile olive oil for *in vivo* work, and used at a concentration of 8 mg/Kg.

### Cell lines and culture conditions

MDA-MB-231 breast cancer cells and ECV-304 endothelial cells were obtained from ATCC and cultured in RPMI-1640 medium supplemented with 10% FBS (Gibco-BRL, UK), 1% of penicillin-streptomycin (100,000 units/L, Gibco-BRL, UK) and incubated at 37 °C in a humidified incubator (95% air, 5% CO_2_).

### *In vitro* proliferation assays

MDA-MB-231 cells were grown to 80% confluency, trypsinized, and plated in duplicate into 6-well plates at a density of 15 × 10^4^ cells. Cells were then incubated for 24 h prior to treatment by Av/OL for 24 h, 48 h or 72 h. For oleamide treatment, cells treated with DMSO served as control. Cells were then harvested and counted with a haemocytometer using the trypan-blue exclusion assay. In parallel, Cell titer 96® Non-Radioactive Cell Proliferation Assay (known as MTT assay, Promega, USA) was also used. Briefly, cells were seeded at a concentration of 1 × 10^4^ cells in triplicate wells for each condition (control, Av, OL or Av/OL). Oleamide was added onto adherent MDA-MB-231 cells. The corrected averages of proliferating cells were determined by subtracting the average reading of RPMI (background measurement) from the averages obtained for control or treatment conditions. The percentage of proliferating cells was determined relative to the number of control cells. Results are expressed as the average of five independent experiments.

### Cell cycle analysis

MDA-MB-231 cells were seeded in duplicate into 6-well plates at 15 × 10^4^ cells and incubated for 24 h prior to drug treatment for 24 h, or 48 h. Cells were then harvested, washed twice with PBS, centrifuged at 200 g for 5 min at 4 °C, re-suspended in 1 mL of cold PBS, fixed in 4 mL of cold absolute ethanol and then stored at −20 °C until staining and analysis. Fixed cells were then treated for 1 h with 200 μM DNase-free RNase A, stained with 1 mM propidium iodide (PI) and incubated for 10 min in the dark. Fluorescence of PI, a measure of DNA content in a cell population, was performed using flow cytometry (FACSCanto II, Becton Dickinson). A total of 10,000 gated events were acquired to assess the proportions of cells in different stages of the cell cycle. Analysis of cell cycle distribution was performed using FlowJo Software.

### Migration, invasion and proliferation RTCA assays

xCELLigence RTCA [A2] DP instrument (Roche, Germany) was used to measure migration, invasion and proliferation. Cells were seeded on a cellular invasion/migration plate (CIM-plate 16) that uses micro-electronic sensors on the underside of an 8 μm microporous polyethylene terephthalate (PET) membrane of a Boyden-like upper chamber. As cells migrate or invade from the upper chamber through the membrane into the bottom chamber, they interact and adhere to the electronic sensors, thus causing an increase in electrical impedance. Changes in the impedance correlate with numbers of migrated or invaded cells on the underside of the membrane, therefore allowing automatic and continuous measurement of migration. For invasion assays, the upper surface of the membrane was precoated with 30 μl of growth factor-reduced Matrigel (BD Biosciences, USA) diluted in serum-free medium at a ratio of 1:20, incubated at 37 °C, 5% CO_2_, for 4 h, then washed with PBS. For migration and invasion assays, 160 μl of RPMI full growth medium was added to the lower chamber of each well (used as a chemoattractant) and 30 μl to the upper chamber, and then the plate was pre-incubated for 1 h at 37 °C. MDA-MB-231 cells were grown in 6-well plates at a density of 15 × 10^4^ cells and incubated for 24 h prior to their treatment, or not, by Av/OL for 48 h. For OL treatment, cells treated with DMSO served as control. Cells were then harvested, counted, resuspended in 120 μl in serum-free media and seeded at a density of 20,000 cells/well in the upper chamber. On the other hand, for on spot treatments, 20,000 untreated cells/well were seeded in the upper chamber, left for 30 min to adhere and then treatment for each condition was added. For proliferation assays, cells were seeded in the same way as above, but in an E-plate and at a density of 7,000 cells/well with an additional 120 μl complete media. Migration, invasion and proliferation were monitored by recording cell impedance every 15 min for a minimum of 18 h.

### Dye-transfer assay

Presence of functional gap junctions was evaluated by flow cytometry using a fluorescent dye-transfer assay, which determines the levels of adhesion and communication between cells. Calcein-AM (Molecular Probes) was used, a membrane permeable fluorescent dye whose AM group is rapidly cleaved by intracellular esterases upon entry into viable cells. The dye (Mwt 600 Daltons) becomes polarized and trapped within the cell, however is able to pass to adjacent cells through functional gap junctions. MDA-MB-231 cells were seeded in 6-well plates at a density of 15 × 10^4^ cells and incubated for 24 h prior to their treatment by Av/OL for 24 h before co-culture. On the next day, cells to be labelled (donor cells) were washed with PBS and incubated in RPMI media for 1 h with 2 μM Calcein-AM. Cells were washed twice with PBS and incubated in serum-free RPMI media for 30 min at 37 °C in order to remove free dye and to allow any non de-esterified dye to leave the cells. Labelled cells (dye donors) were then co-cultured with unlabelled cells (dye acceptors) at a ratio of 1:1 at 37 °C for the indicated incubation times, for MDA-MDA or MDA-ECV. Co-cultures were trypsinized, fixed in 4% formaldehyde and analyzed by flow cytometry. The increase in MFI of unlabeled cells reflected increased dye transfer through gap junctions. Labelled and unlabelled cells without any treatment were used as control. Data was analysed by FlowJo software.

### RNA extraction

Total RNAs from cultured cells were isolated using NucleoSpin^®^ RNA II Kit according to manufacturer’s instructions (Macherey-Nagel, USA). The quantity of RNA was measured using the NanoDrop® ND-1000 UV-Vis Spectrophotometer. RNA purity was assessed using the absorbance ratio of 260 to 280 nm, where a value of 1.8–2.0 indicated good quality RNA. Finally, RNAs were treated by Deoxyribonuclease I (Fermentas, USA) according to manufacturer’s instructions followed by RNA precipitation and quantification.

### Real time PCR

To quantify differences in mRNA expression, DNA synthesis was monitored using Syber Green as a dye which fluoresces upon binding to dsDNA, but not ssDNA. Human specific GAPDH primers (h-G6PDH Housekeeping Gene Set, Roche Applied Science, Germany) were used as control. During RT step, ssDNA was synthesized from 1 μg RNA, using Revert Aid 1^st^ Strand cDNA synthesis kit (Fermentas, USA), followed by PCR. The 20 μl reactions were incubated in a CFX96 real-time PCR system (Bio-Rad, Germany). The reaction includes 1 μL RNA, 7.5 μL mixing hybridization of RNA, 0.5 μL of each primer (20 μM), 1 μL probes (4 μM), 1.3 μL Mn (OAC)_2_ and water. The conditions were 95 °C for 10 min, then 45 cycles each of denaturation at 95 °C for 15 sec, annealing at 60 °C for 15 sec and extension at 72 °C for 15 sec. Results were analysed by Light Cycler Software version 3. Also, 1 μl of cDNA was loaded in duplicate with forward and reverse primers of our gene of interest at a concentration of 5 μM, and mixed with Syber Green. Primers used were the following: GAPDH, F: TGGTGCTCAGTGTAGCCCAG and R: GGACCTGACCTGCCGTCTAG; Cx26, F: CCTCCCGACGCAGAGCAA and R: CAGACAAAGTCGGCCTGCTCA; Cx43, F: TCCCTCCAGCAGTTGAG and R: CTTCACTACTTTTAAGCAAAAGAG; HIF-1α, F: AGCCAGATCTCGGCGAAGT and R: CAGAGGCCTTATCAAGATGCG; VEGF, F: AGGCCCACAGGGATTTTCTT and R: ATCAAACCTCACCAAGGCCA; CXCR4, F: CCTCCTGCTGACTATTCCCGA and R: GGAACACAACAACCCACAAGT. GAPDH, VEGF and CXCR4 had an annealing temperature of 55 °C, while it was 62, 52, and 58 °C for Cx26, Cx43 and HIF-1α; respectively. Negative control (water without DNA) was used to check for contamination. Normalized fold expression relative to the control was calculated and plotted by CFX-Biorad manager to compare differential expression.

### Protein extraction and quantification

Proteins from cultured cells were homogenized in lysis buffer containing protease and phosphatase inhibitors, followed by sheering with a 27 gauge needle. Protein concentrations were then determined using “DC Protein Assay II” kit (Bio-Rad, Germany).

### Western Blotting

Protein samples were loaded into the wells of stacking gel and run until bromophenol blue reached the bottom of the gel. Gels were then transferred to PVDF membranes at 4 °C at 30–40 volts overnight. The membrane was then blocked in 5% fat-free milk, prepared in Tween PBS, for 1 h at RT. Detection of the protein of interest was achieved by probing the membrane with the primary antibody of interest. Primary antibodies used were monoclonal antibodies for Cx43, Cx26, and GAPDH (Sigma, USA), the latter used to ensure equal loading of samples. Molecular weight markers enabled the determination of protein sizes. Intensity of bands was then determined by densitometry, using ImageJ software.

### Gelatin zymography

Proteins extracted from cultured MDA-MB-231 cells or from lung tissues of MDA-MB-231 injected mice (please see below) were run along with FBS (positive control) on 0.75 mm SDS-PAGE gel, containing gelatin as a substrate, washed twice and incubated overnight in substrate buffer at 37 °C with shaking. Gels were then stained with coomasie blue for 1 h at RT, de-stained with ethanol, acetic acid and water solution. Band staining intensity was determined by densitometry, using ImageJ software.

### Xenograft mouse model of solid cancer metastasis

This study was approved by the Institutional Animal Care and Utilization Committee (IACUCC# 10-07-154) of the American University of Beirut. Mice were obtained from Jackson Laboratory (Bar Harbor, USA) and housed under pathogen-free conditions with constant temperature and humidity control. Six to eight weeks old immune-deficient NSG mice (NOD.Cg-*Prkdc*^scid^ *Il2rg*^tm1Wjl^/SzJ) were injected either subdermally (*s.d.*) or intravenously (*i.v.*) with 2 × 10^6^ or 1 × 10^6^ MDA-MB-231 cells; respectively. Briefly, cells were washed twice, re-suspended in incomplete media and filtered in a cell strainer (40 μm) to remove clumps. Cells were then injected either *s.d.* into the subcutaneous area of the neck region or *i.v.* into the blood stream, using the tail-vein. The first model (*s.d.* injection) allows the development of solid tumor into the site of injection, so a primary tumor forms which metastasizes later on. However, metastases in the second model (*i.v.* injection) mimic blood-borne cancer cell adhesion to ECs of organs and extravasation. Mice were randomized at the day of tumor cell injection. Groups included control, Av, OL, or Av/OL. Avastin (10 mg/Kg) or oleamide (8 mg/Kg) were administered intraperitonealy (*i.p*.), for 4 weeks, twice and three times per week, respectively. Oleamide was prepared from 5 mg/1 mL filtered olive oil solution, dissolved for 1 h in a sonicator, diluted and injected.

### Survival analysis and tumor volume measurement

Animals were monitored on a daily basis until they died; the date of death recorded. Survival curves were plotted using Kaplan-Meier method on Graphpad Prism 5. Tumor growth and progression were monitored by measurement of tumor size with a calliper device, on a weekly basis. Tumor volume (V, mm^3^) was determined by the equation: V = π/_6_ abc (a = length; b = width; c = height)^12^. Data was plotted as the average tumor size of a minimum of 15 mice per treatment group.

### Animal tissue harvesting

Mice were anesthetized with isofurane then euthanized by cervical dislocation either at weeks 5, 7, 9 or at weeks 4, 5, 6, 7 for *s.d.* or *i.v.* injected mice; respectively. Lung and liver samples were obtained and divided into 2 parts: the first was fixed in formalin and embedded in paraffin for immune-histochemistry; the second was snap frozen in liquid nitrogen then stored at −80 °C to be used later for protein analysis and zymography.

### Tissue fixation, paraffin embedding and haematoxylin and eosin staining (H&E)

Tissues were fixed in 10% formalin in a biopsy cassette for 2 days at RT, and then dehydrated. Before staining, slides were deparaffinised and rehydrated. The sections were then mounted on glass slides and stained in haematoxylin and eosin. Histology of sections was then examined under a light microscope.

### Statistical Analysis

Results are expressed as individual data or as the mean ± SEM. Statistical comparisons were performed using the Student’s t-test in order to determine statistical significance. The *p* value was determined and values for p < 0.05, p < 0.001, p < 0.0001 (*, **, ***; respectively) were considered significant. Microsoft Excel and GraphPad software were used to perform statistical analysis.

### Compliance with Ethics Guidelines concerning Animal Rights:

This study was approved by the Institutional Animal Care and Utilization Committee (IACUCC# 10-07-154) of the American University of Beirut. All experiments were conducted in compliance with current Good Clinical Practice standards and in accordance with relevant guidelines and regulations and the principles set forth under the Declaration of Helsinki (1989).

### Results

#### *In vitro* study

##### OL or the combination of Av/OL reduce the viability of MDA-MB-231 cells

OL is a gap junction inhibitor whose anti-metastatic potential and mechanism of action are still not well understood. The non-cytotoxic concentrations of Av/OL (50 μM/20 μM) on MDA-MB-231 cells were determined prior to any experiments (Data not shown).

**Figure 1:**
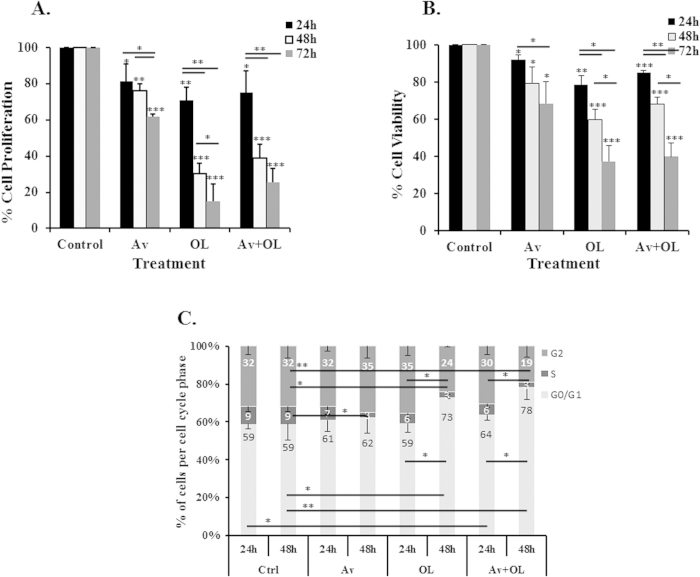
OL and Av/OL reduce the viability and induce cell cycle arrest of MDA-MB-231 breast cancer cells. (**A**) The % of growth inhibition of Av/OL was calculated relative to the untreated cells by trypan-blue exclusion assay, or (**B**) MTT viability assay. (**C**) Histogram analysis showing the percentage of cell cycle distribution of MDA-MB-231 treated cells after 24 h or 48 h, relative to controls. The percentage of each cycle was determined using FlowJo software. Four or five separate experiments were performed for each time point and treatment condition, reported as the mean plus or minus the standard error of the mean. *, **, *** indicate p < 0.05, p < 0.001, p < 0.0001; respectively.

The effect of Av/OL on the proliferation of MDA-MB-231 cells was measured using trypan-blue exclusion assay. Avastin showed a modest, but significant, 20% decrease on proliferation of MDA-MB-231 cells at 24 h or 48 h of treatment whereas it significantly reduced by 40% the proliferation of cells at 72 h, compared to untreated cells (Fig. 1A). On the other hand, OL attenuated the growth of MDA-MB-231 cells by 30%, 70% or 85%, after 24 h, 48 h or 72 h of treatment; respectively. Finally, when Av and OL were applied in combination, the inhibition was similar to what was obtained using OL alone, without any synergistic effect (Fig. 1A). Those results were confirmed using the MTT assay, which gave a similar cell viability profile (Fig. 1B). The inhibitory effect of OL on cell proliferation was fully reversible since normal growth was restored after removal of the drug.

##### OL and Av/OL induce cell cycle arrest in the G_1_/S phase of MDA-MB-231 cells

To confirm whether the inhibition of cell proliferation was due to cell cycle arrest, cell cycle analysis was performed at 24 h, or 48 h of treatment with Av, OL or their combination. Flow cytometry analysis of cellular DNA content distribution on MDA-MB-231 cells showed that, at 24 h, only the combined Av/OL treatment induced significant changes in the G_0_/G_1_ phases (p < 0.05), relative to the control (Fig. 1C). However, G_0_/G_1_ and G2 phases were significantly increased after 48 h of treatment with OL (p < 0.05) or Av/OL (p < 0.001), but not Av. Indeed, OL caused the accumulation of the cell population in the G_0_/G_1_ phases and the loss of cells from G2 phase. The combination treatment at 48 h’s resulted in more significant differences in both G0/G1 and G2 phases, which were statistically significant in comparison to the results at 24 h’s (p < 0.05). Meanwhile, all treatment conditions (Av, OL or Av/OL) resulted in a significant decrease in the S phase at 24 h, which was further decreased at 48 h (Fig. 1C). This indicates that treatment with OL or Av/OL, but not Av, diminished the number of cycling cells and induced an arrest in G_1_/S phase, at 48 h of treatment.

##### OL and Av/OL significantly reduce migration and invasion of MDA-MB-231 cells

Real Time Cell Analysis (RTCA) was used in order to measure the proliferation, migration, and invasion of MDA-MB-231 cells, following Av/OL treatment. During RTCA, interaction of cells with gold electrode is correlated to impedance which is reported as cell index. Two different treatment conditions were used, “48 h” or “on spot”. In the first condition, MDA-MB-231 cells were seeded and treated for 48 h, which was followed by their trypsinization and seeding in the RTCA E-plate to assess their proliferation, migration and invasion. In contrast, “on spot” treatment was performed by culturing the MDA-MB-231 cells in the E-plate while initiating the treatment after their adhesion. The rationale for using the two conditions was to measure any difference in the effect of 48 h pre-treatment with Av/OL on cells, in comparison to the direct effect of “on spot” treatment.

**Figure 2:**
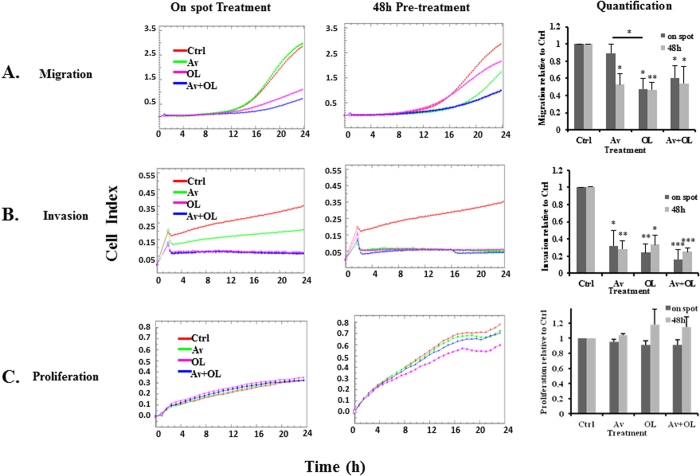
OL and Av/OL significantly reduce migration and invasion of MDA-MB-231 cells, by Real-Time Cell Analysis (RTCA) assay. (**A**) Migration, (**B**) invasion and (**C**) proliferation plots of cell index vs time for on spot treatment of Av/OL (Left panels), or 48 h pre-treatment with Av/OL (Middle Panels). Red corresponds to Ctrl, green to Av, pink to OL and blue to Av + OL. Quantification graphs (Right panels) of normalized cell index values, relative to controls, for both on spot treatment and 48 h pre-treatment. Cell impedance readings were taken every 15 min for a minimum of 18 h. Results are representatives of three independent experiments (n = 3). *, **, ***indicate p < 0.05, p < 0.001, p < 0.0001; respectively.

The on spot treatment of Av had no effect on migration whereas a 50% significant decrease was found at 48 h post-treatment (p < 0.05, Fig. 2A). On the other hand, on spot and 48 h treatment by OL or Av/OL reduced by >50% the migration of the cells (p < 0.05, Fig. 2A). On the other hand, there was a 70%–80% significant decrease in invasion for on spot or 48 h post-treatment by Av, OL or Av/OL; respectively (p < 0.05, p < 0.001 or p < 0.0001; Fig. 2B). It’s worth noting that invasion of MDA-MB-231 cells decreased slightly at 24 h of OL treatment, but not Av (data not shown). The effect of Av/OL on migration and invasion assays, for both on spot and 48 h treatments, was not due to the increase of cell number since the proliferation curves (total cell numbers) for those RTCA experiments was not modified (Fig. 2C).

##### OL and Av/OL significantly reduce homo and hetero-cellular communications of MDA-MB-231 cells, by dye transfer assay

**Figure 3:**
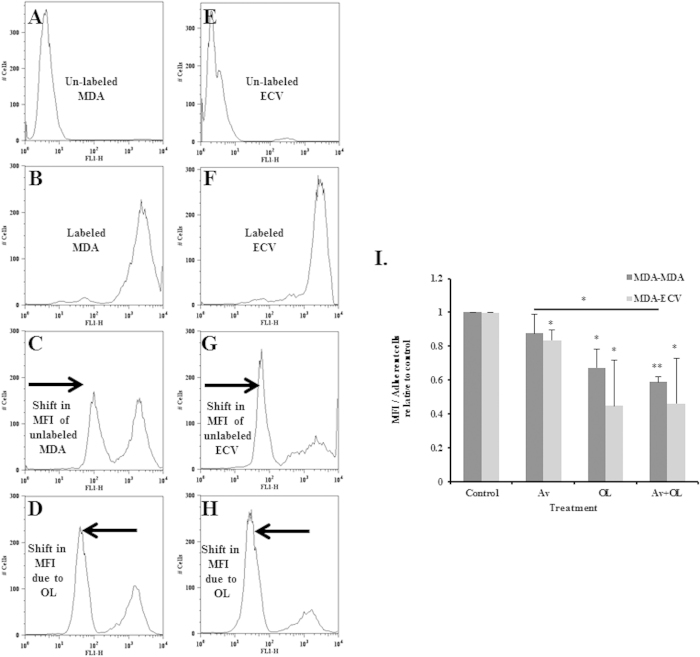
OL significantly reduces homo and hetero-cellular communications of MDA-MB-231 cells, by dye transfer assay. (**A**) Unlabeled MDA-MB-231 cells (**B**) Calcein-labelled MDA-MB-231 cells (**C**) Unlabeled MDA-MB-231 cells co-cultured with calcein-labelled MDA-MB-231 cells for 1 h, in the absence of any drug treatment (**D**) MDA-MDA cells pre-treated with oleamide for 24 h. (**E**) Unlabeled ECVs (**F**) Calcein-labelled MDA-MB-231 cells (**G**) Unlabeled ECVs co-cultured with calcein-labelled MDA-MB-231 cells for 30 min, in the absence of any drug treatment (**H**) MDA-ECV pre-treated with oleamide for 24 h. (**I**) Quantification of cell communication within MDA-MB-231 cells (dark) or between MDA-MB-231 cells and ECVs (light). Quantification was done by measuring the mean fluorescent intensity (MFI) using FlowJo software. Results are representatives of three independent experiments (n = 3), reported as the mean plus or minus the standard error of the mean. *, **, ***indicate p < 0.05, p < 0.001, p < 0.0001; respectively.

Calcein labeled MDA-MB-231 cells were co-cultured with unlabeled MDA-MB-231 cells for 1 h, in order to test for homo-cellular communication. In another set of experiments, calcein labeled MDA-MB-231 cells were co-cultured with unlabeled ECVs for 30 min, in order to test for hetero-cellular communication (Fig. 3). Unlabeled MDA-MB-231 cells were pre-treated for 24 h. The shift of mean fluorescence intensity (MFI) was the highest in the control cells and was considered as 100%. Flow cytometry results showed significant effect of OL on GJIC, whether between MDA-MB-231 cells themselves (homo-cellular interaction, Fig. 3D) or between MDA-MB-231 cells with ECVs (hetero-cellular interaction, Fig. 3H). Indeed, OL showed a significant decrease of >50% in cellular communication (p < 0.05, Fig. 3I). It’s worth noting that Av had no effect on GJIC when MDA-MDA cells were studied whereas it caused a slight decrease in GJIC (~15%) when MDA-ECVs were used. Combination treatment of Av/OL showed similar results to OL alone, confirming that Av has little effect on GJIC (Fig. 3). This inhibition of gap junctions leads to a decrease of GJIC between cancer cells, and between cancer and endothelial cells.

##### Av/OL significantly down-regulated transcriptional expression of metastatic (VEGF, HIF-1α, and CXCR4) and connexin markers (Cx26 and Cx43) in MDA-MB-231 cells

**Figure 4:**
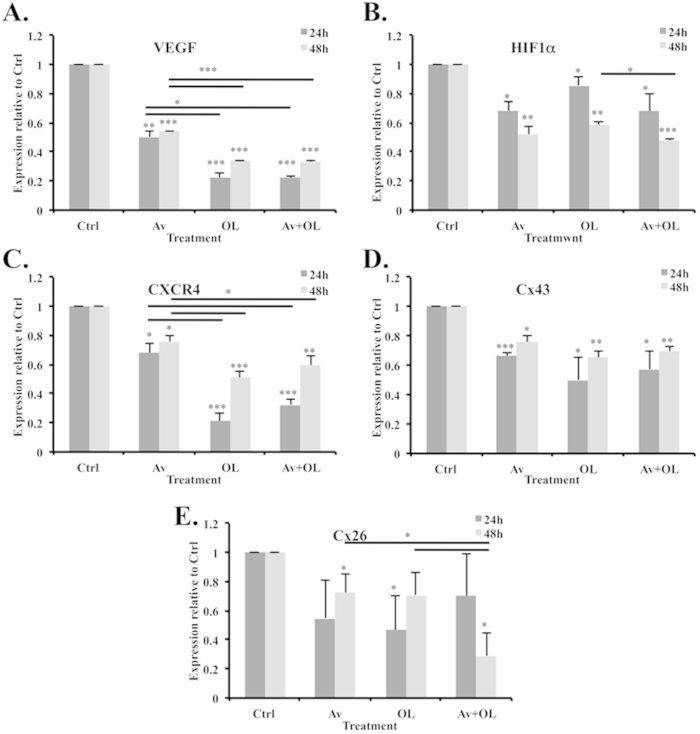
Av/OL reduces transcriptional expression of metastatic (VEGF, HIF-1α, and CXCR4) and connexin markers (Cx43 and Cx26) in MDA-MB-231 cells. (**A**) Real-Time PCR showing the effect of Av/OL treatments in MDA-MB-231 cells on human VEGF mRNA expression, (**B**) HIF1α, (**C**) CXCR4, (**D**) Cx43, and (**E**) Cx26. Data on each target mRNA was normalized to GAPDH. Results are representatives of three independent experiments. *, **, ***indicate p < 0.05, p < 0.001, p < 0.0001; respectively.

MDA-MB-231 cell line is highly invasive since it highly expresses VEGF, HIF-1α, CXCR4, MMP2, Cx43 and Cx26 (Supp Fig. 1). To investigate the anti-metastatic and anti-angiogenic potential of Av/OL at the transcriptional level, we examined the expression of human VEGF, HIF-1α, CXCR4, Cx43, and Cx26 transcripts by real time PCR, normalized to GAPDH internal control. Results showed that Av decreased VEGF expression (p < 0.001), whereas OL and Av/OL decreased VEGF levels more significantly, at both 24 h and 48 h (p < 0.0001, Fig. 4A). Moreover, Av, OL and Av/OL showed very similar decrease on HIF-1α expression at both 24 h and 48 h (p < 0.05 and p < 0.001; respectively, Fig. 4B). As for CXCR4, Av treatment showed a slight decrease in its expression whereas both OL and Av/OL showed a very significant decrease at 24 h and 48 h post-treatment (p < 0.0001 and p < 0.001; respectively, Fig. 4C). Furthermore, Av, OL or Av/OL decreased significantly, by approximately 2-fold, the expression of Cx43 at 24 h and 48 h (Fig. 4D). Finally, the decrease illustrated for Cx26 was significant mainly at 48 h for Av, OL or Av/OL treatment (Fig. 4E), but also at 24 h for OL alone. Therefore, we suggest that Av/OL, by the possible action on selective metastatic markers, could inhibit paracrine interactions and GJIC between tumor and surrounding cells.

##### OL reduces Cx43, Cx26 and MMP9 protein expression levels in MDA-MB-231 cells

**Figure 5:**
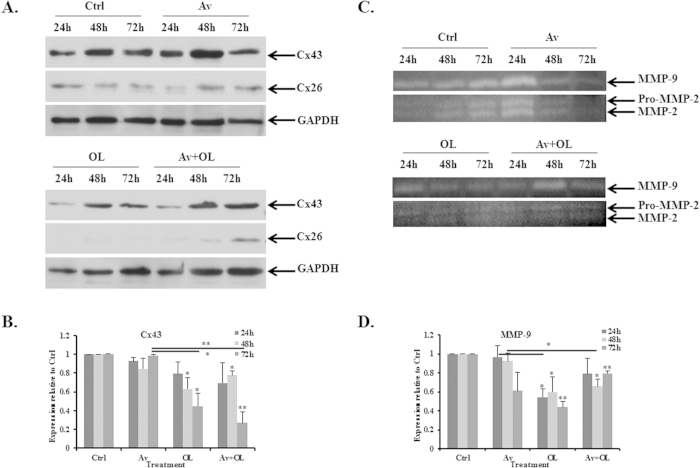
OL reduces Cx43, Cx26 and MMP9 protein expression levels in MDA-MB-231 cells. (**A**) Western blot for Cx43, Cx26 and GAPDH from Av/OL treated MDA-MB-231 cells, at 24 h, 48 h or 72 h. (**B**) Quantification analysis of Cx43 western blots. Values represent the average fold change of Cx43 expression, normalized to GAPDH, and relative to control, for a total of 3 western blots (n = 3). (**C**) Gelatin zymography on Av/OL-treated MDA-MB-231 cells. FBS was used as an internal control (not shown). Proteins were migrated on a gel containing gelatin, the substrate of MMPs, in order to assess the activation status and levels of these enzymes. (**D**) Quantification analysis of gelatin zymography showing the effect of treatment on control, Av, OL or Av/OL treated cells for MMP-9 levels. The intensity of each band was determined by densitometry, using ImageJ software. Quantification of each band was normalized to controls. Western blots were also normalized to GAPDH. Results are representatives of three independent experiments (n = 3). *, **, ***indicate p < 0.05, p < 0.001, p < 0.0001; respectively.

Protein expression levels of Cx43 and Cx26 were then investigated by western blot. Data showed that Av did not affect Cx43 or Cx26 protein expression levels at any time point. However, OL decreased Cx43 levels by 40–60%, at 48 h and 72 h post-treatment (p < 0.05, Fig. 5A,B). Furthermore, data showed that OL, but not Av, decreased very significantly Cx26 levels, at all time points (Fig. 5A). Finally, Av/OL combination treatment was efficient in reducing Cx26 levels at 24 h and 48 h, but not 72 h post treatment. On the other hand, combination treatment of Av/OL gave similar results to OL alone, for both Cx43 and Cx26, whereas Av did not have any synergistic effect, even at 72 h of treatment (p > 0.05). These results on OL confirm the transcriptional expression data.

Gelatin zymography was performed in order to detect the enzymatic activity of MMP-9 in Av/OL-treated MDA-MB-231 cells. Results showed the presence of the active forms of both MMP2 and MMP-9, which correlates with the proteolytic activity of tumor cells implicated in invasion (Fig. 5B). Data showed that Av treatment decreases active MMP-9 levels by 40% at 72 h only (Fig. 5C,D). On the other hand, OL significantly reduced MMP-9 levels by more than 2-folds, at 24 h, 48 h or 72 h (Fig. 5C,D). Finally, Av treatment did not have any synergistic effect with OL.

#### *In vivo* study

##### Effect of Av/OL on tumor volume and survival of MDA-MB-231 injected NSG mice

**Figure 6:**
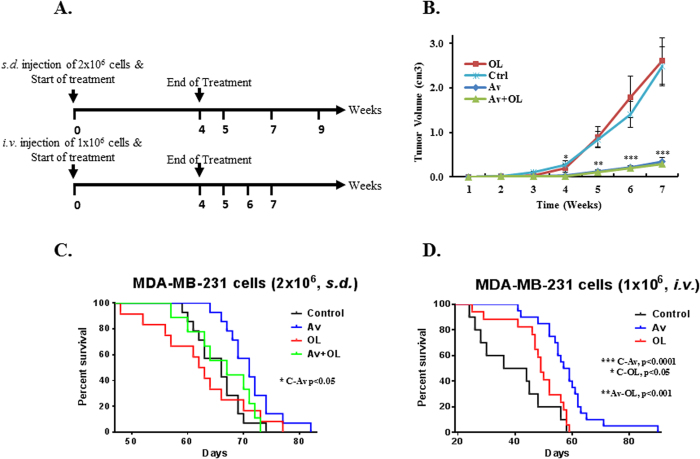
Av/OL increases the survival of intra-venously injected NSG mice and reduces tumor volume of sub-dermally injected mice. (**A**) *In vivo* mouse models used to test the effect of Av (10 mg/Kg) or OL (8 mg/Kg). Briefly, 2 × 10^6^ or 1 × 10^6^ MDA-MB-231 cells were used for *s.d.* (under the dorsal neck region) or *i.v.* injection of NSG mice; respectively. Numbers indicate the time points for measurements or tissue sampling. (**B**) Growth of the primary tumor was monitored by measuring its dimensions on a weekly basis. Tumor volume (cm^3^) was determined as described in materials and methods. Data was plotted as the average tumor size of a minimum of 15 mice, per treatment group, in 3 separate experiments. Error bars represent the standard error of the means (SEM). (**C**) Survival curves of sub-dermally injected NSG mice followed by Av/OL treatment. (**D**) Survival curves of intra-venously injected NSG mice followed by Av/OL treatment.

We assessed the effect of Av/OL on metastasis *in vivo*, using 2 experimental mouse models and treatment strategies (Fig. 6A). We first determined the number of MDA-MB-231 cells to be used for sub-dermal *(s.d.)* or intra-venous *(i.v.)* injection (Supp Fig. 2). Av/OL treatment of *s.d*. or *i.v.* injected NSG mice was not toxic as assessed by weight measurements, behaviour, macroscopic and microscopic morphology of internal organs (data not shown). Furthermore, we investigated the *in vivo* anti-tumor activity of Av/OL in sub-dermally injected MDA-MB-231 mice. Tumor volume was delayed and significantly reduced by Av or Av/OL treatment at 4, 5, 6, and 7 weeks, as compared to untreated animals (***: p < 0.0001, Fig. 6B). On the other hand, OL treatment did not show any regression of tumor growth, at all time-points.

Data showed that NSG mice injected subdermally with 2 × 10^6^ MDA-MB-231 cells had a better survival rate when treated with Av, in comparison to control untreated mice (Fig. 6C, *: p < 0.05). However, OL or the combination of Av/OL did not show any significant improvement in the survival rate, in comparison to controls (Fig. 6C). On the other hand, NSG mice injected intravenously with 1 × 10^6^ MDA-MB-231 cells had a better survival rate when treated with avastin (***: p < 0.0001) or oleamide (*: p < 0.05), in comparison to untreated MDA-MB-231 injected mice (Fig. 6D). Indeed, 50% of Av-treated mice survived beyond 60 days, time at which all control, but also OL-treated, MDA-MB-231 injected mice had already died. Finally, the survival rate of Av-treated *i.v.* injected mice was significantly higher than those treated with OL (**: p < 0.001 between Av and OL).

##### Effect of Av/OL on metastasis to the lung and liver of MDA-MB-231 injected NSG mice

**Figure 7:**
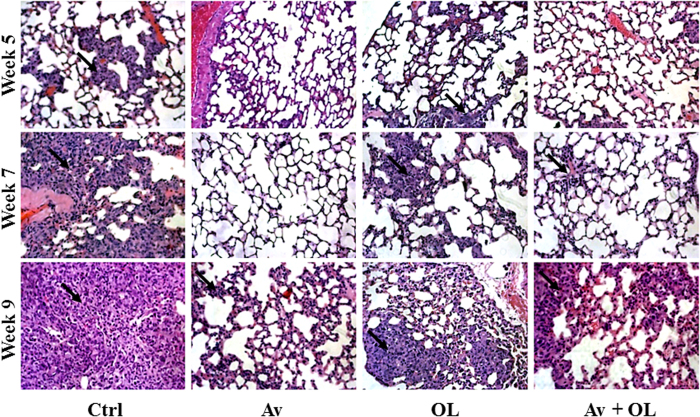
Avastin, but also oleamide, inhibits metastasis to the lungs of sub-dermally injected MDA-MB-231 mice. To examine the degree of tumor cell metastasis, H&E staining of *s.d.* injected MDA-MB-231 cells into NSG mice was performed on the lungs. Metastasis was studied by histology, after 5, 7 or 9 weeks of tumor cell injection and beginning of treatment. Arrows indicate infiltration. All pictures were taken at 32X.

The effect of Av/OL on survival and tumor volume correlated to reduced metastasis to the lungs and livers of *s.d.* injected MDA-MB-231 mice. Infiltration of tumor cells was observed in the lungs of untreated control mice at 5 weeks, which increased considerably with time to reach its highest level at 9 weeks (Fig. 7). On the other hand, no infiltration was observed in the lungs of mice treated by Av or Av/OL at 5 or 7 weeks, but started to appear at 9 weeks. Moreover, very rare infiltration was observed in the lungs of OL-treated mice at 5 weeks which increased with time to reach its highest level at 9 weeks, but always considerably less than control mice.

**Figure 8:**
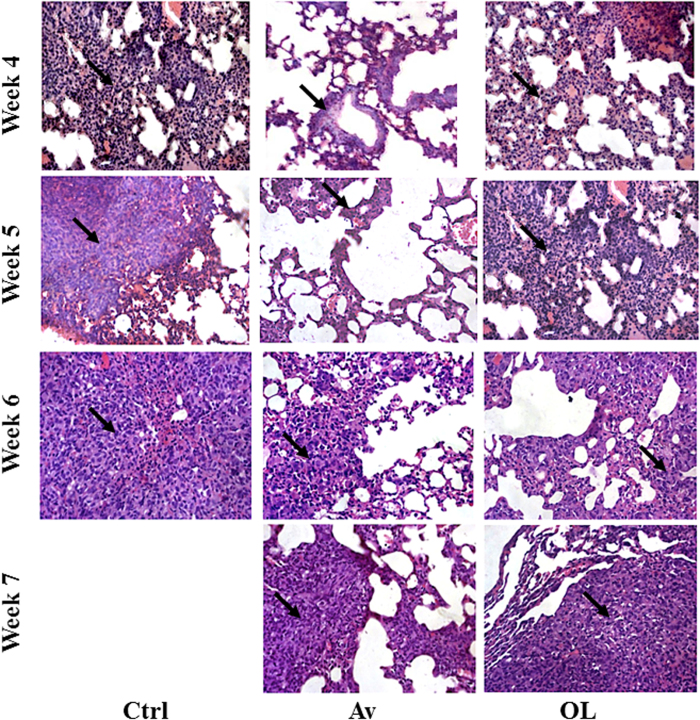
Avastin, but to a lesser extent oleamide, inhibits metastasis to the lungs of intra-venously injected MDA-MB-231 mice. H&E staining of *i.v.* injected MDA-MB-231 cells into NSG mice was performed on the lungs. Following treatment, lungs were sampled to examine the extent at which the cells had metastasized, at 4, 5, 6 or 7 weeks. Arrows indicate infiltration. All pictures were taken at 32X.

Metastasis to the lungs of *i.v.* injected MDA-MB-231 untreated mice appeared very early (week 4) which increased dramatically over time (weeks 5 and 6) until the mice died from excessive tumor infiltration before week 7 (Fig. 8). In contrast, there was no or relatively low infiltration in the lungs of Av-treated mice, at weeks 4 and 5, which became more prominent towards weeks 6 and 7, and mice were still alive. Furthermore, OL treatment reduced the infiltration to the lungs at weeks 4 and 5, but to a lesser extent at later stages (weeks 6 and 7); in comparison to controls (Fig. 8) and OL-treated mice were still alive. Therefore, there was a direct correlation between survival rate and reduced metastasis to the lungs of *i.v.* injected MDA-MB-231 mice.

The liver, another common secondary site of cancer metastasis, was also examined for tumor cell infiltration in *s.d.* injected mice. Liver metastatic foci were detected in untreated mice only after week 7 (Supp Fig. 3). However, mice that were treated with Av, OL or Av/OL showed attenuated metastasis to the liver. On the other hand, liver infiltration in *i.v.* injected untreated mice started at week 5 and increased dramatically at week 6, before their death shortly after (Supp Fig. 4). However, there was no or rare infiltration in the livers of Av-treated mice, at week 5 or 6, but became evident at week 7. Finally, OL treatment had modest reduction in the infiltration to the livers at weeks 5, 6, or 7, compared to controls (Supp Fig. 4).

In summary, there was a significant difference in the lungs and livers of Av-, but also OL-treated mice, in comparison to control mice, at all intervals of time. These observations indicate that Av reduces the metastasis of MDA-MB-231 cells to the lungs and livers of *s.d.* injected mice. Finally, OL also reduces the metastasis to the lungs and livers of *i.v.* injected mice, but to a lesser extent than Av. This data suggests that metastasis occurs less efficiently in OL-treated mice, in comparison to untreated mice. These results correlate positively with an increased survival rate for OL-treated *i.v*.-injected mice.

##### OL reduces MMP9 protein expression levels in the lungs of MDA-MB-231 *i.v.* injected NSG mice

**Figure 9:**
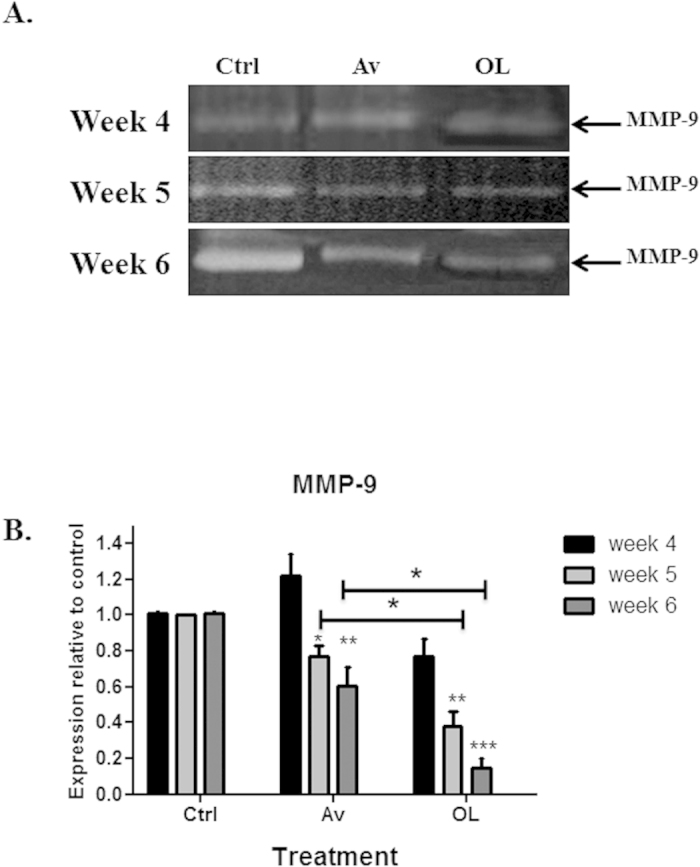
OL reduces MMP9 protein expression levels in the lungs of intra-venously injected MDA-MB-231 mice. (**A**) Gelatin zymography on lungs of Av or OL treated mice, injected intravenously with MDA-MB-231 cells. Treatment with Av, but also more significantly with OL, decreased MMP-9 levels at 5 and 6 weeks post-treatment. (**B**) Quantification analysis of gelatin zymography showing the effect of treatment on control, Av, or OL treated mice. The decrease in MMP-9 levels between Av and OL treatment was significant for weeks 5 and 6 (p < 0.05). The intensity of each band was determined by densitometry, using ImageJ software. Quantification of each band was normalized to controls. Results are representatives of three independent experiments (n = 3). *, **, ***indicate p < 0.05, p < 0.001, p < 0.0001; respectively.

Gelatin zymography was performed in order to detect the enzymatic activity of MMP-9 in Av or OL-treated mice, previously injected with MDA-MB-231 cells intravenously. Results showed the presence of the active form of MMP-9, which correlates with the proteolytic activity of tumor cells implicated in extravasation (Fig. 9A). Data showed that Av treatment decreases MMP-9 levels by only a modest 20–30% at weeks 5 or 6 post-treatment (Fig. 9A,B). On the other hand, OL significantly reduced MMP-9 levels by more than 60–80%, at those time points (Fig. 9A,B). It’s important to note that the decrease in MMP-9 levels between Av and OL treatment was significant for weeks 5 and 6 (p < 0.05), which further proves the effect of OL treatment *in vivo*.

### Discussion

Metastasis is the hallmark of malignant tumors. Despite recent advances in diagnosis and therapeutic modalities of cancer, there is little improvement in overall survival of patients with metastasis. Intricate interaction between tumor cells and their microenvironment is pivotal in the pathogenesis of metastasis^13^. Hence, when targeting cancer, cells should be considered in the context of their complex milieu, including hetero-cellular interactions^14^,^15^. Angiogenesis inhibitors^16^, as a class of anti-neoplastic agents, are of interest since their main targets are ECs. We proposed and demonstrated a role for both VEGF^17^ and direct cancer cell-endothelial cell communication in the extravasation process^18^,^19^ and stressed that targeting cancer cell extravasation is a viable treatment option^20^,^21^. Such therapy is thought to inhibit retraction of ECs by inhibiting the communication between cancer cells and ECs, thus reducing the metastatic colonization of tumor cells into secondary organs.

In this study, we investigated the anti-angiogenic, anti-tumor and anti-metastatic activities of Avastin (Av)^22^, an anti-VEGF antibody; and Oleamide (OL)^23^,^24^,^25^, a gap junction inhibitor, using MDA-MB-231 human breast cancer cells *in vitro* and a xenograft murine model *in vivo*. We showed that gap junction inhibition by Av/OL reduce MDA-MB-231 breast cancer cell invasion and metastasis *in vitro* and *in vivo*. We observed a significant time-dependent inhibition of MDA-MB-231 cell growth using OL, but not Av. Although the specificity of Oleamide inhibition of gap junctions is of broad nature, it has been used successfully in multiple *in vitro* and *in vivo* studies to elucidate the roles of gap junctions^26^,^27^,^28^. Furthermore, OL or Av/OL treatment, but not avastin, diminished the number of cycling cells and induced an arrest in G_1_/S phase, at 48 h of treatment. Growth inhibitory effects might be also due to apoptosis since extensive net decrease in total number of treated cells was observed at 72 h of treatment. Consistent with our results, previous studies showed that Av induces only minor cell cycle perturbations in bladder cancer cell lines and that suppression of VEGF expression in metastatic cells *in vitro* induced their apoptosis, and inhibited the constitutively elevated PI3K activity, an important factor for their survival^29^. The effect of OL in arresting the MDA-MB-231 cell cycle progression is supported by studies that describe a role for connexins in cell cycle dynamics where Cx43 was shown to modulate the expression of several cell cycle genes, including cyclin A, cyclin D1, cyclin D2, and cyclin-dependent kinases, p21 and p27^30^.

In addition, OL and Av/OL significantly reduced the migration and invasion of MDA-MB-231 cells. OL showed a significant decrease in GJIC on homo- and hetero-cellular communications of MDA-MDA or MDA-endothelial cells, respectively. A significant decrease in the transcriptional expression of metastatic markers (VEGF, HIF-1α, and CXCR4) and connexins (Cx26 and Cx43) was also observed^10^ at 24 h and 48 h of treatment of Av/OL. OL, as expected, decreased the expression of Cx43, but also Cx26, protein levels which suppresses invasion as cited in a recent article where silencing of Cx43 suppressed invasion, migration and metastasis in lung hepatocellular carcinoma cells^31^. In addition, Av and OL decreased the protein expression levels of MMP-9, as assessed by zymography. Av and OL treatment elicited a decrease in both invasion and migration potential of cancer cells, especially for Av/OL, which showed a synergistic decline in invasiveness at both “on spot” and 48 h post-treatment. VEGF is a potent angiogenic activator, overexpressed in most human tumors and whose increased expression correlates with tumor progression^32^. VEGF promotes signaling pathways that result in EC activation, migration, proliferation and the formation of a pre-metastatic niche^33^. In turn, activated endothelial cells release factors such as MMPs, which have the ability to degrade the ECM. The latter process forms a prerequisite for the retraction, proliferation and migration of ECs toward the tumorigenic mass and neovascularization. The new vessels provide cancer cells with nutrients and a low resistance pathway into the bloodstream and potentially the ability to invade other organs^34^. In tumor cells, VEGF up-regulates the secretion of proteases that degrade the BM, and increases the expression of adhesion molecules, hence mediating interaction with tumor cells^35^. MMP-2 and MMP-9 expression is correlated with higher invasive phenotypes and are considered as potential targets to treat cancer^36^. They also regulate invasion and migration by degrading BM components, particularly by cleaving laminin-5, IL6R, TGF and TNF^37^. Data on VEGF and HIF1α decreased expression emphasize the anti-angiogenic potential of Av and illustrate a role of OL in angiogenesis and subsequent migration and intra/extravasation. The decrease in CXCR4 highlights its role in promoting migration and proliferation of tumor cells which is consistent with previous results where its inhibition decreased the invasion in human colorectal cancer^38^. Additionally, MMP-9 down-regulation is associated with a decrease in invasion of tumor cells and this has been shown in a previous study that elucidate the blockage of fibroblast-dependent skin cancer invasion and reduction in vascularization due to MMP inhibition^39^.

In the *in vivo* assays, tumor cells were injected either sub-dermally into the lower neck region where a primary tumor forms or intravenously where tumor cells are injected into the bloodstream from the tail vein. Metastases in the latter model form more rapidly and in greater numbers. This model assesses the post-intravasation stage and addresses events at the level of extravasation and beyond. Av, but not OL, reduced tumor size of NSG mice injected sub-dermally with MDA-MB-231 cells, which was also associated with increased survival of Av-treated mice. On the other hand, Av but also OL, separately, significantly increased the survival of intravenously (*i.v.*) injected mice where Av-treated mice had a better survival curve than those treated with OL. Moreover, in comparison to controls, independent treatments of Av or OL alone reduced pulmonary and hepatic metastatic foci in *s.d.* or *i.v.* injected mice *in vivo*, although to a lesser extent in the *s.d.* model than in the *i.v.* model, which also showed a better survival rate. Finally, the combination treatment of Av/OL, which was only assessed in the *s.d.* model, also showed a decrease in pulmonary and hepatic metastatic foci, but very similar to the treatment by Avastin alone. In conclusion, while Av has anti-angiogenic, anti-tumor and anti-metastatic activities, OL has anti-metastatic activity, presumably at the extravasation level, providing further evidence for the role of GJIC in cancer cell extravasation. Finally, it’s important to note that we have demonstrated in this study that OL resulted in the down-regulation of MMP-9 in the lungs of *i.v.* injected mice, which is associated with a decrease in invasion of tumor cells and might be responsible for the reduction in vascularization^39^.

A function for gap junctions and connexins in cancer metastasis is not novel. Connexins have been shown to act as tumor suppressor genes in breast cancer since a long time^40^,^41^. Several studies performed in our lab and others have implicated homocellular and heterocellular communication in metastasis^42^,^43^,^44^,^45^,^46^,^47^. The notion that connexin expression is spatio-temporally regulated has been shown earlier^48^ and is actually emphasized by our work. This study was devised to test our earlier *in vitro* observation that tumor cells extravasate through a transient angiogenesis-like mechanism^7^ where a cooperation between intercellular communication and paracrine action of VEGF enhanced tumor cell extravasation.

We show that targeting gap junctions can attenuate intercellular communication, migration, invasion, and metastatic dissemination. Our data provide evidence that OL exhibit unique interesting anti-metastatic effects against breast cancer cell lines *in vitro* and *in vivo*, most likely via the reduction of Cx26 and Cx43, but also possibly HIF-1α, VEGF, CXCR4 and other downstream signaling cascades. Finally, OL treatment resulted in significant reduction in secondary site invasion of both s.d.- and i.v.-injected NSG mice, but only a significantly increased survival of i.v.-injected mice.

This study highlighted the role of intercellular communication in extravasation. Although Avastin have been retracted from breast cancer treatment modalities, combination therapies that include Avastin are still employed clinically in different cancer types^49^,^50^,^51^. Avastin had favorable effects on both our *in vivo* models (s.d. and i.v.) where its effect on the extravasation process was translated into more than two weeks survival advantage. In a clinical setting, the use of first line treatment that include Avastin may be enhanced in the presence of gap junction inhibitors to attenuate blood borne cancer cell extravasation.

Overall, this study confirms that extravasation of tumor cells across the endothelial interface couldn’t be ascribed to a single factor. Indeed, there is a dynamic interplay between paracrine signaling, most notably that of VEGF, and hetero-cellular communication that ultimately leads to extravasation. Consequently, this study has set the background for further investigation of functional consequences and the underlying biochemical events that occur between tumor and endothelial cells at the extravasation site. Finally, Avastin, classically known for its anti-angiogenic effects, and Oleamide, a gap junction inhibitor, both display significant inhibitory effects on tumor extravasation, a notion that provides an attractive option as part of a multi-disciplinary approach to cancer management.

### Additional Information

**How to cite this article**: Zibara, K. *et al.* Anti-angiogenesis therapy and gap junction inhibition reduce MDA-MB-231 breast cancer cell invasion and metastasis *in vitro* and *in vivo. Sci. Rep.* **5**, 12598; doi: 10.1038/srep12598 (2015).

## Supplementary Material

Supplementary Information
