# Supplementary material for: Anti-angiogenesis therapy and gap junction inhibition reduce MDA-MB-231 breast cancer cell invasion and metastasis in vitro and in vivo
Source: Sci Rep. 2015 Jul 28;5:12598. doi: 10.1038/srep12598 (PMC4517444; doi:10.1038/srep12598)

Anti-angiogenesis therapy and gap junction inhibition reduce MDA-MB-231 breast cancer cell invasion and metastasis *in vitro* and *in vivo*.

Kazem Zibara[1,*], Zahraa Awada[1], Leila Dib[2], Jamal El-Saghir[2], Sara Al-Ghadban[2], Aida Ibrik[1], Nabil El-Zein[1], and Marwan El-Sabban[2,*].

Supplementary Figure Legends

Supp Figure 1: mRNA expression profile of metastatic markers in MDA-MB-231 cells.

mRNA expression by RT-PCR of some metastasis-related markers showed that this cell line is highly aggressive and invasive since it highly expresses VEGF, HIF-1α,, CXCR4, MMP2, Cx26 and Cx43.

Supp Figure 2: Survival curves of NSG mice injected with various doses of MDA-MB-231 cells.

(A) Survival curves of sub-dermally (*s.d.*) injected NSG mice with $2x10^6$ or $5x10^6$ MDA-MB-231 cells.

(B) Survival curves of intra-venously (*i.v.*) injected NSG mice with $1x10^6$ or $5x10^6$ MDA-MB-231 cells.

Supp Figure 3: Avastin, but also oleamide, showed attenuated metastasis to the livers of sub-dermally injected MDA-MB-231 mice.

H&E staining of *s.d.* injected MDA-MB-231 cells into NSG mice was performed on the livers. Metastasis was studied by histology, after 5, 7 or 9 weeks of tumor cell injection and beginning of treatment. Arrows indicate infiltration. All pictures were taken at 32X.

Supp Figure 4: Avastin, but to a lesser extent oleamide, inhibits metastasis to the livers of intra-venously injected MDA-MB-231 mice.

H&E staining of *i.v.* injected MDA-MB-231 cells into NSG mice was performed on the livers. Following treatment, livers were sampled to examine the extent at which the cells had metastasized, at 4, 5, 6 or 7 weeks. Arrows indicate infiltration. All pictures were taken at 32X.

Supp Figure 1

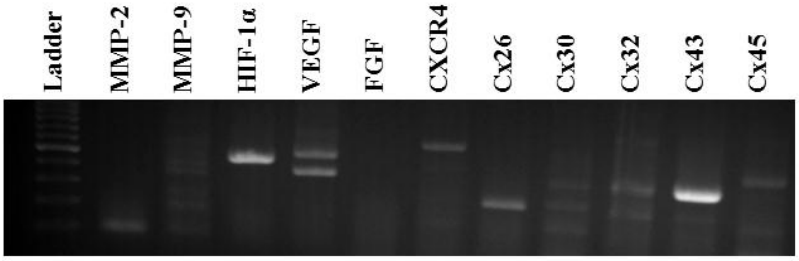

Supp Figure 2

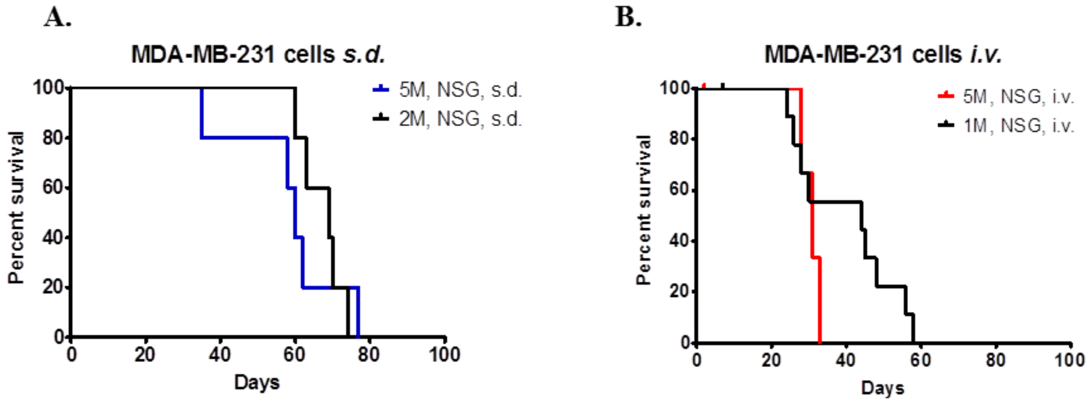

Supp Figure 3

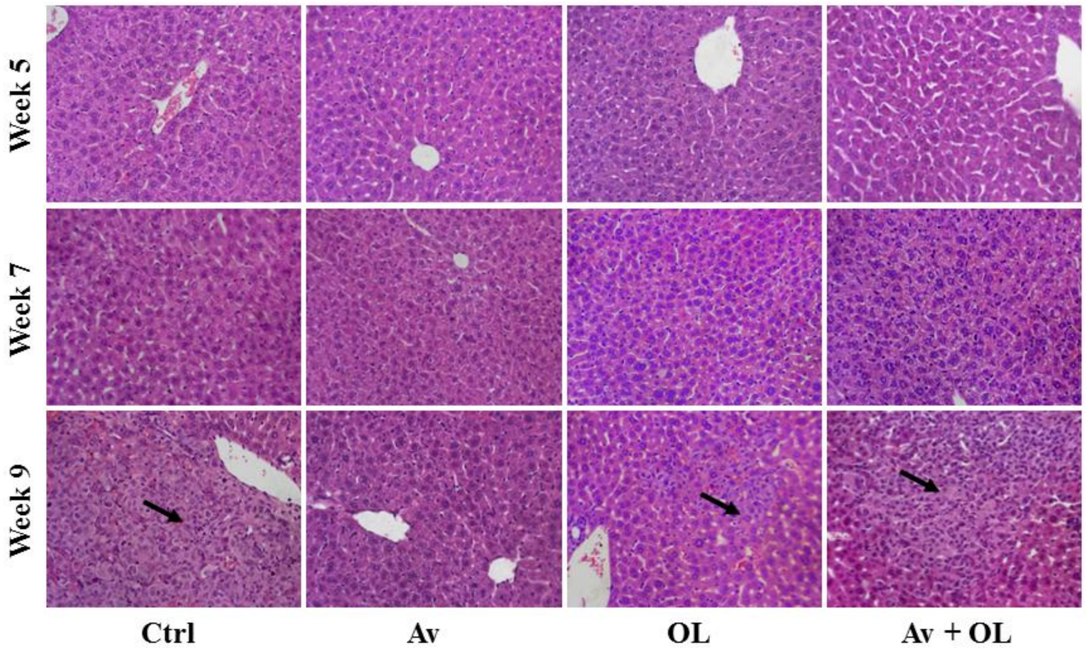

Supp Figure 4

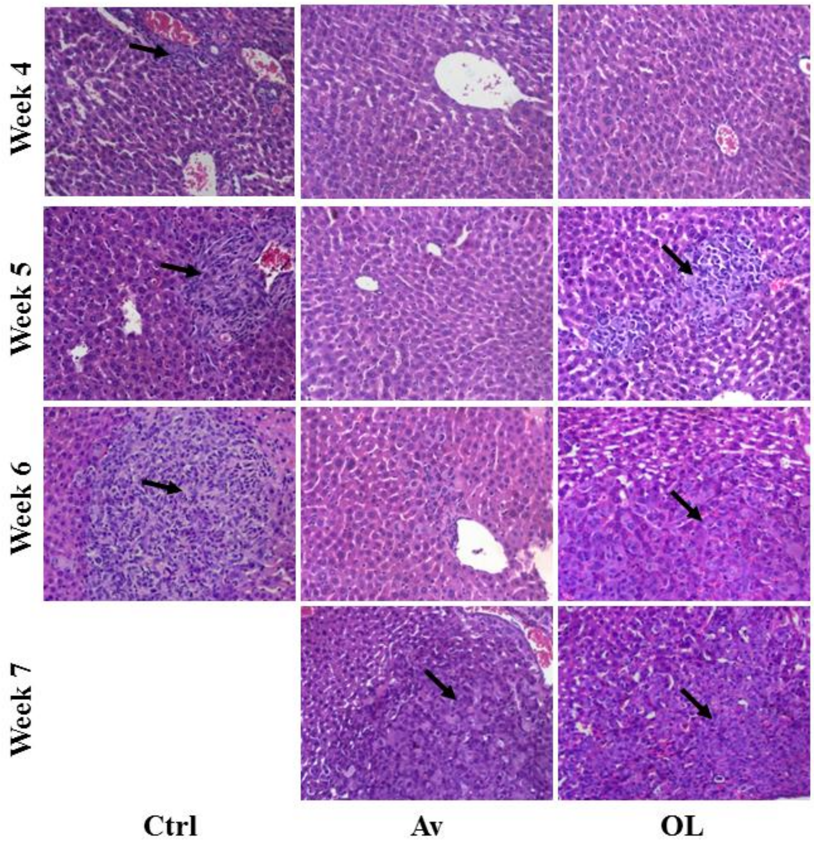

Supplement: Supplementary Information [file srep12598-s1.pdf]
